# ﻿Two new varieties of *Agapetes* (Ericaceae) from Xizang, China

**DOI:** 10.3897/phytokeys.249.133820

**Published:** 2024-11-08

**Authors:** Yi-Hua Tong, Xiang-Long Guo, Bing-Mou Wang, Zi Wang, Yong-Jie Guo

**Affiliations:** 1 Key Laboratory of Plant Resources Conservation and Sustainable Utilization & Key Laboratory of Digital Botanical Garden of Guangdong Province, South China Botanical Garden, Chinese Academy of Sciences, Guangzhou, Guangdong, 510650, China South China Botanical Garden, Chinese Academy of Sciences Guangzhou China; 2 South China National Botanical Garden, Chinese Academy of Sciences, Guangzhou, Guangdong, 510650, China South China National Botanical Garden, Chinese Academy of Sciences Guangzhou China; 3 School of Life Sciences, Peking University, Beijing, 100871, China Peking University Beijing China; 4 Panyu Central Hospital, Guangzhou, Guangdong, 511402, China Panyu Central Hospital Guangzhou China; 5 College of Fungi and Ecology, Fujian Agriculture and Forestry University, Fuzhou, Fujian, 350002, China Fujian Agriculture and Forestry University Fuzhou China; 6 Germplasm Bank of Wild Species, Kunming Institute of Botany, Chinese Academy of Sciences, Kunming, Yunnan, 650201, China Kunming Institute of Botany, Chinese Academy of Sciences Kunming China; 7 University of Chinese Academy of Sciences, Beijing, 100049, China University of Chinese Academy of Sciences Beijing China

**Keywords:** Medog County, morphology, taxonomy

## Abstract

Two new varieties from Xizang, China, i.e. Agapetesinterdictavar.flaviflora and A.forrestiivar.parvifolia, are described and illustrated. Agapetesinterdictavar.flaviflora differs from the nominate variety in having yellow and smaller corollas with shorter lobes and anthers with shorter appendages at the base. Agapetesforrestiivar.parvifolia is distinguished from the nominate variety in the smaller leaves with an acute apex, nearly transverse secondary veins and puberulent peduncle. Taxonomic notes on these taxa are also provided.

## ﻿Introduction

The genus *Agapetes* D. Don ex G. Don (Ericaceae-Vaccinioideae-Vaccinieae) comprises ca. 115 species worldwide, with 63 distributed in China (Fang and Stevens 2005; Tong 2014; POWO 2024; Yang et al. 2024). General overviews of the historic taxonomic studies of this genus in China were presented by Tong (2016) and Tong et al. (2019). Xizang Autonomous Region, with 42 species and one variety including the recently published *A.huangiana* Bin Yang, Y. H. Tan & Y. H. Tong and *A.rhuichengiana* Bin Yang & Y. H. Tan, harbours the most species of *Agapetes* in China (Huang 1986; Fang and Stevens 2005; Tong 2014; Yang et al. 2022, 2024). Most *Agapetes* species from Xizang are concentrated in Medog County, which possesses diverse topography as well as high biodiversity. Nowadays, as road access to Medog is improving, scientists have more opportunity to conduct explorations in this area. New species of plants, fungi and animals have been continuously discovered in Medog County in recent years (e.g. Tong et al. (2021); Song et al. (2022); Yang et al. (2022, 2024); Pei et al. (2024)). During two recent field trips to Medog, the authors encountered undescribed variation within two species of *Agapetes*, which is described as two new varieties and illustrated below.

## ﻿Materials and methods

Specimens were collected from Medog County, Xizang Autonomous Region, China during two field expeditions in January 2020 and April 2024, respectively. Descriptions were based on both field observations and dried specimens. The studied specimens were mainly deposited at the Herbaria of Institute of Botany, Chinese Academy of Sciences (PE), Kunming Institute of Botany, Chinese Academy of Sciences (KUN) and South China Botanical Garden, Chinese Academy of Sciences (IBSC). Measurements were performed with a ruler and small plant parts were observed and measured under a stereomicroscope (Mshot-MZ101). The concepts of infraspecific ranks follow Christensen (1987).

## ﻿Taxonomic treatment

### ﻿1. Agapetes interdicta

Taxon classificationPlantaeEricalesEricaceae

(Hand.-Mazz.) Sleumer in Bot. Jahrb. Syst. 70: 106. 1939.

08C78204-434D-565D-886D-D462D2C950D8

≡ Pentapterygiuminterdictum Hand.-Mazz. in Anz. Kaiserl. Akad. Wiss. Wien, Math.-Naturwiss. Kl. 60: 186. 1923. Type: China, Yunnan, above Schutsche on the Taron [Dulongjiang River, upper course of the Irrawaddy], 2400–2800 m elev., 9 Jul 1906, *H. F. v. Hand.-Mazz. 9465* (holotype: W, barcode no. WU0044070, image!; isotypes: W, image!, E, barcode no. E0078234, image!).
= Agapetesinterdictavar.stenoloba (W. E. Evans) Sleumer in Bot. Jahrb. Syst. 70: 106. 1939. ≡ Pentapterygiuminterdictumvar.stenolobum W. E. Evans in Notes Roy. Bot. Gard. Edinburgh 15: 207. f. 1A. 1927. Type: Myanmar, Salween-Kiu Chiang [Dulongjiang River] divide, 3050–3350 m elev., Jul 1924, *G. Forrest 25678* (lectotype: E, barcode no. E00078236, image!, designated here; isoloectotypes: K, barcode no. K000357906, image!, P, barcode no. P06672383, image!); remaining syntype: ibid., Oct. 1924, *G. Forrest 25802* (K, barcode no. K000357907, image!).

#### Description.

Evergreen shrub, 0.3–0.8 m tall, epiphytic on trees, with inflated root tubers. Twigs angled, 1–3 mm in diam., pubescent and glandular-setose, glabrescent when old. Leaves alternately scattered, often pseudo-whorled; petiole 1–2 mm long, glabrous, glaucous; blades leathery, oblanceolate to elliptic, 2–4.5 × 0.8–2.3 cm, glabrous, mid-veins conspicuously raised on both sides, secondary veins 7–9 pairs, at an angle of 40–50° with the mid-vein, conspicuous and raised adaxially, slightly raised abaxially, veinlets conspicuous and raised adaxially, inconspicuous abaxially, base cuneate, with 1 basal gland each side at a distance of 1.5–3 mm away from the junction of blade and petiole, margin slightly revolute when dry, nearly entire, except the apex with 2–3 serrulas on each side, each serrula terminated with a gland, apex acute or apiculate. Inflorescences shortly racemose, 1–3(–7)-flowered, often cauline, sometimes also axillary; peduncle 1–7 mm long, puberulent, with 2 or 3 sterile bracts; floral bracts ovate-triangular, ca. 0.7 × 0.6 mm, margin entire, apex acute; pedicels 3–11(–13) mm long, densely puberulent, sometimes intermixed scattered shortly glandular setose, apex slightly expanded; bracteoles 2, basal, similar to floral bracts, but smaller, deciduous. Hypanthium glabrous or sparsely pubescent at base; tube 2–4 mm long, 4–5.5 mm wide (including wings), sparsely pubescent, conspicuously winged, wings to 1 mm wide; limb 4.5–6 mm long, glabrous, nearly divided to 2/3, lobes narrowly ovate-triangulate to ovate, 4–10 mm long, apex apiculate. Corolla red, rarely yellow-green or yellow, with 4–5 inconspicuous V-shaped veins, tubular, 5-angled, 1.3–3(–3.4) cm long, glabrous; lobes slightly reflexed, greenish or yellow, triangular-subulate to triangular, 2–9 mm long. Stamens 10; filaments flat, 2–5 mm long, glabrous; anthers 1.2–2.6(–3.2) cm long, thecae echinate, each locule with a very small appendage at the bottom, appendages 0.2–1.1 mm long, tubules 2–4× as long as thecae, without spurs on the back. Style slender, as long as the corolla or slightly exerted; stigma truncate; ovary 10-pseudoloculed, each locule with several ovules; disc glabrous. Fruit stalk densely puberulent; berry ca. 1 cm in diam., subglabrous, with persistent calyx lobes and wings.

#### Distribution and habitat.

Southeast Xizang and northwest Yunnan of China and north Myanmar. It grows on the tree trunks under evergreen forests at elevations of 2300–2700(–2900) m.

#### Phenology.

Flowering in March to April; fruiting in August.

### ﻿1a. Agapetes interdicta var. interdicta

Taxon classificationPlantaeEricalesEricaceae

A47DC21C-C6F5-57EB-A39B-ED03B90E1575

#### Description.

Corolla usually red, rarely yellow-green, 2.2–3 cm long, lobes 6–9 mm; anther appendages ca. 1.1 mm long.

#### Distribution and habitat.

Southeast Xizang and northwest Yunnan of China and north Myanmar. It grows on the tree trunks under evergreen forests at elevations of 2300–2700(–2900) m.

#### Phenology.

Flowering in March to April; fruiting in August.

#### Examined specimens.

China. Yunan: Gongshan County, foot of Gaoligong Mountains, 1960 (without date), *Su-Kung Wu s.n.* (KUN, barcode no. 0230889) • [Gongshan County], Taron-Taru Divide [Gaoligong Mountains], Tehgai to Ahtehmai; 2300 m elev.; 30 October 1938; *Te-Tsun Yu 20890* (KUN, barcode no. 0034482, PE, barcode no. 01907994) • Gongshan County, Gaoligong Mountains; 8 March 2023 (fl.), *Yi-Hua Tong, Jing-Bo Ni, Bing-Mou Wang, Wei-Hao Pan TYH-2615* (IBSC) • Gongshan County, Dulongjiang, Erduibei; 2300 m elev.; 16 May 1991; *Dulongjiang Expedition 6839* (KUN, barcode no. 0231130) • Gongshan County, Dulongjiang, Maku; 2000 m elev.; 16 December 1990 (fl. bud); *Dulongjiang Expedition 1117* (KUN, barcode nos. 0231128 & 0231131), *1118* (KUN, barcode nos. 0231124 & 0231125) • ibid.; 8 March 1991 (fl.), *Dulongjiang Expedition 4269* (KUN, barcode nos. 0231122 & 0231123), *4280* (KUN, barcode no. 0231133) • Gongshan County, Dulongjiang, Qiawudang; 2650 m elev.; 25 March 1991; *Dulongjiang Expedition 492*6 (KUN, barcode no. 0230893), *4986* (KUN, barcode nos. 0230890 & 0230891), *4987* (KUN, barcode no. 0231126) • Gongshan County, Dulongjiang, Sandui; 2550 m elev.; 27°34′N, 98°21′E; 11 September 1979 (fr.); *Hong Wang 64917* (HITBC) • Gongshan County, Dulongjiang, Xishaofang; 3200 m elev.; 30 March 1991; *Dulongjiang Expedition 5387* (KUN, barcode nos. 0231134 & 0231135). Xizang: Medog County, near Hanmi; 1900 m elev.; August 1974 (fr.); *Qianghai-Xizang Expedition s.n.* (KUN, barcode no. 0230892) • Zayu County, Delei Valley; 28°10′N, 96°30′E; 7000–8000 ft elev.; 12 April 1924 (fl.); *F. Kingdon-Ward 8082* (K, barcode no. K000639646, image). Myanmar. Kachin State: Kangfang; 7000–9000 ft elev.; 9 January 1939 (fl.); *F. Kingdon-Ward 206* (NY, barcode no. 02651412, image) • Top of Hpare Vally; 8000 ft elev.; 5 April 1938 (fl.); *C. W. D. Kermod 17166* (K, barcode no. K000639647, image) • Tzi-tzo-ti; 26°58′N, 98°28′E; 7000–8000 ft elev.; May 1925; *G. Forrest 26585* (K, barcode no. K000639648, image).

### ﻿1b. Agapetes interdicta var. flaviflora

Taxon classificationPlantaeEricalesEricaceae

Y.H.Tong, B.M.Wang & X.L.Guo var. nov.

CE6ACB71-6A57-5751-AC76-67E2D4F7C26A

urn:lsid:ipni.org:names:77351464-1

Fig. 1

#### Type.

China • Xizang Autonomous Region: Medog County, Beibeng Xiang, De’ergong Village; 2300–2700 m elev.; 3 April 2024 (fl.); *Xiang-Long Guo TYH-2898* (holotype IBSC, isotype PE).

**Figure 1.**
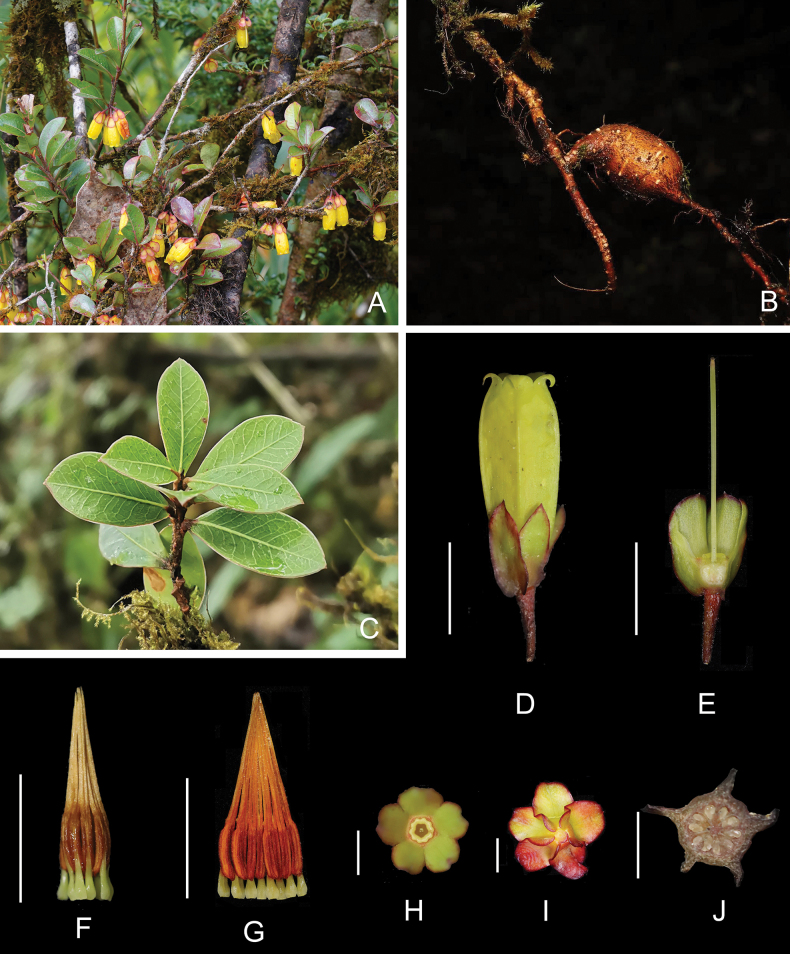
Agapetesinterdictavar.flaviflora**A** flowering plant **B** root tuber **C** leafy branch **D** flower **E** longitudinal section of flower with corolla and stamens removed to show style and ovary **F** androecium, abaxial view **G** androecium, adaxial view **H** calyx and disc, top view **I** calyx, bottom view **J** transection of ovary. Scale bars: 1 cm (**D–G**); 5 mm (**H–I**); 3 mm (**J**). **A**, **B**, **E**, **G** and **I** by Zi Wang, others by Xiang-Long Guo.

#### Diagnosis.

This new variety differs from the nominate variety in having a yellow and smaller (1.3–1.8 cm) corolla with shorter (2–2.5 mm) lobes and anthers with shorter (ca. 0.2 mm) appendages at the base.

#### Etymology.

The variety epithet is derived from its striking yellow flowers. The Chinese name is given as 黄花中型树萝卜(Chinese pinyin: huáng huā zhōng xíng shù luó bo).

#### Distribution and habitat.

This species is currently known only from the type locality, i.e. Medog County, Xizang, China. It grows on the tree trunks under broadleaved forests at elevations of 2300–2700 m.

#### Phenology.

The new variety flowers in March to April.

#### Taxonomic notes.

**Figure 2.**
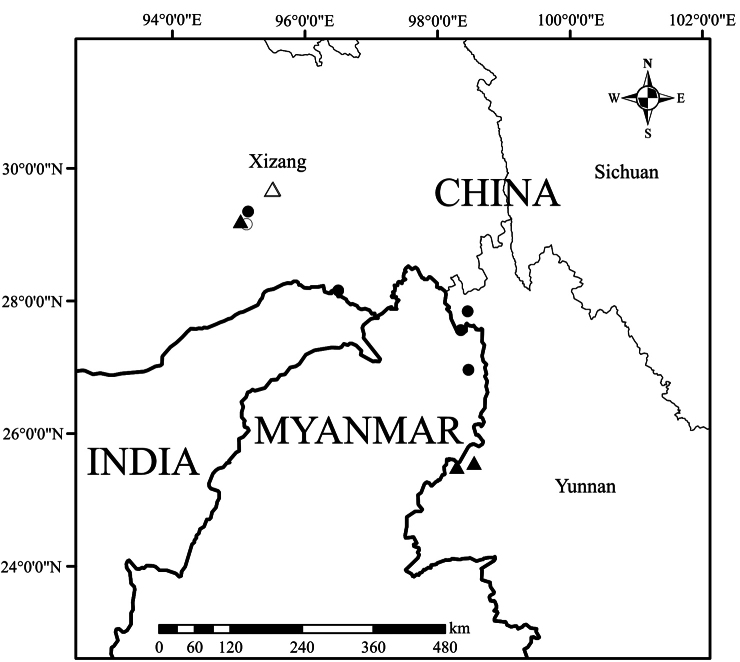
Distribution map of Agapetesinterdictavar.interdicta (black circle), A.interdictavar.flaviflora (white circle), A.forrestiivar.forrestii (black triangle) and A.forrestiivar.parvifolia (white triangle).

**Figure 3.**
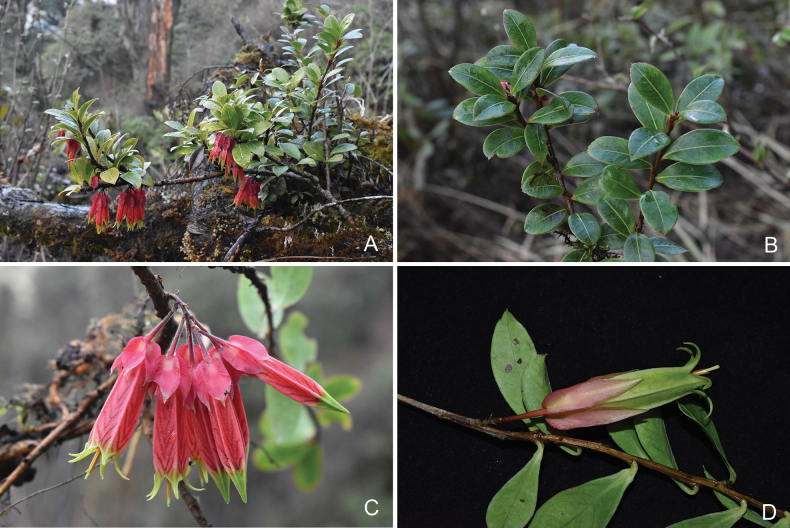
Agapetesinterdictavar.interdicta**A** flowering plant **B** leafy branch **C** inflorescence **D** variation of yellow-green corolla. All by Yi-Hua Tong. **A–C** from Gongshan County of Yunnan Province **D** from Tengchong City of Yunnan Province.

Compared to this new variety, the nominate variety, Agapetesinterdictavar.interdicta, has a much wider distribution including southeast Xizang and northwest Yunnan of China, and north Myanmar (Fang and Stevens 2005) (Fig. 2). Although the two varieties differ markedly in the colour, size and lobes of corollas, their vegetative parts are nearly the same so that it is difficult to identify them if the material is sterile. It is worth noting that Agapetesinterdictavar.interdicta also rarely bears yellow-green flowers, but the differences in corolla size and lobes remain consistent (Fig. 3). The same kind of corolla colour variation also occurs in other *Agapetes* species, such as *A.hosseana*, which usually bears red or orange flowers, but some populations have green flowers (Watthana 2015). Except for the corolla colour variation, the differences between this taxon and *A.interdicta* are mainly presented in some quantitative characters, viz. the size of corollas and the length of appendages at the base of anthers, which seem not significant enough to differentiate them as distinct species. Thus, we recognise this taxon (A.interdictavar.flaviflora) as a new variety rather than a new species. Another variety with narrower calyx lobes, A.interdictavar.stenoloba (W. E. Evans) Sleumer, was merged with the nominate variety by Fang and Stevens (2005). We agree with this treatment because the shapes of calyx lobes of this species can vary from narrowly laceolate to ovate even in a population, such as the Dulongjiang population. A lectotype for this name was designated here incidentally, since two collections were cited in the protologue without clear type designation (Evans 1927). Due to the striking yellow corollas, A.interdictavar.flaviflora has high ornamental value.

### ﻿2. Agapetes forrestii

Taxon classificationPlantaeEricalesEricaceae

W. E. Evans in Notes Roy. Bot. Gard. Edinburgh 15: 202. t. 220. 1927.

6BD1C89B-962C-5BA5-9587-8E91A79F59C9

#### Type.

China. Yunnan: Lung-fan [Long Fang]; 25°34′N, 98°33′E; May 1925, *G. Forrest 26583* (holotype E, barcode no. E00078232, image!; isotypes: K, barcode no. K000357892, image!, NY, barcode no. 00008156, image!, PE, barcode no. 00195226!).

#### Description.

Evergreen shrub, 0.25–1 m tall, epiphytic on trees, with inflated root tubers. Tubers globose or spindle-like. Twigs slightly angled, 1–2 mm in diam., densely pubescent and sparsely setose. Leaves spirally scattered, glabrous; petiole 0.5–5 mm long; leaf blades leathery, ovate to ovate-lanceolate, 1.1–4 × 0.5–1.2 cm, glabrous, mid-veins conspicuously raised on both sides, secondary veins 2–3 pairs, nearly transverse, strongly impressed adaxially, slightly raised abaxially, veinlets strongly impressed adaxially, inconspicuous abaxially, base rounded, without basal glands, margin slightly revolute when dry, each side with 5–7 serrulae, each serrula terminated with a gland, apex acute. Inflorescences corymbose, (2–)3–10-flowered, axillary; peduncle slender, 0.8–2 cm long, glabrous or puberulent, with several sterile bracts; floral bracts ovate-triangular, ca. 0.5 × 0.5 mm, margin serrulate, apex acute; pedicels 7–15 mm long, nearly glabrous, slightly expanded upwards; bracteoles 2, basal, similar to floral bracts, but smaller, deciduous. Hypanthium nearly cupular, 0.9–4.5 × 1–3.5 mm, glabrous; limb 1.5–2 mm long, glabrous, nearly divided to base, lobes triangular to narrowly triangular, 1.5–2 × 0.8–2 mm, glabrous, apex acute. Corolla carmine, with 5–6 zig-zag transverse veins, green at the apex, tubular, 5-angled, 9–20 × 2.5–5 mm, glabrous; lobes green, slightly reflexed, ovate-triangular, 0.8–2 mm long. Stamens 10, 9–15 mm long; filaments flat, 1–1.8 mm long, pubescent; anthers 8–13 mm long, thecae 1.5–5.5 mm long, echinate, each locule with a very small appendage at the bottom, tubules 6–8 mm long, each with an oblique pore ca. 3.5 mm long, without spurs on the back. Style slender, 1.2–1.9 cm long, glabrous; stigma truncate; ovary 10-pseudoloculed, each locule with several ovules; disc glabrous. Mature fruit white, more or less with crimson dots, subglobose.

### ﻿2a. Agapetes forrestii var. forrestii

Taxon classificationPlantaeEricalesEricaceae

AC98EFAA-172D-5670-83FD-0258D67DDF58

#### Description.

Leaf blades 3–4 × 0.8–1.2 cm, apex acuminate, secondary veins arcuate. Peduncle glabrous. Corolla ca. 2 cm long.

#### Distribution and habitat.

Southeast Xizang and west Yunnan of China and north Myanmar. It grows on the tree trunks under evergreen forests at elevations of 1800–2700 m.

#### Phenology.

Flowering in December to May of next year.

#### Examined specimens.

China. Yunnan: Tengchong City, Langya Mountain, 2200–2400 m elev.; 14 April 1984; *Wen-Zheng Li & Yin-Qing Guo 8411* (SWFC). Xizang Autonomous Region • Medog County, Beibeng Xiang, Xirang, De’endong; 2300–2400 m elev.; 29 April 1983 (fl.); *Bo-Sheng Li & Shu-Zhi Cheng 4396* (PE, barcode nos. 01907990 & 01907988) • ibid., Xirang, Sangxing; 2300 m elev.; 26 April 1983 (fl.); *Bo-Sheng Li & Shu-Zhi Cheng 4705* (PE, barcode no. 01907985) • ibid., Xirang, Xidengshan; 2300 m elev.; 1 May 1983 (fl.); *Bo-Sheng Li & Shu-Zhi Cheng 4770* (PE, barcode no. 01907989). Myanmar. Kachin State: Near Panwa Pass; 7000–9000 ft elev.; 11 March 1939 (fl.); *F. Kingdon-Ward 388* (NY, barcode nos. 02651408 & 02651409, image).

### ﻿2b. Agapetes forrestii var. parvifolia

Taxon classificationPlantaeEricalesEricaceae

Y.H.Tong & B.M.Wang var. nov.

8B920CDB-C499-553E-998F-D6244048E853

urn:lsid:ipni.org:names:77351465-1

Fig. 4

#### Type.

China • Xizang Autonomous Region: Medog County, Damu Xiang, Km 80 on Zhamo Road, epiphytic on trees in evergreen broad-leaved forest; 29°40'59.9"N, 95°30'6.3"E; 2191 m elev.; 3 January 2020 (fl.); *Yi-Hua Tong & Bing-Mou Wang TYH-2363* (holotype IBSC, isotypes IBSC, PE).

**Figure 4.**
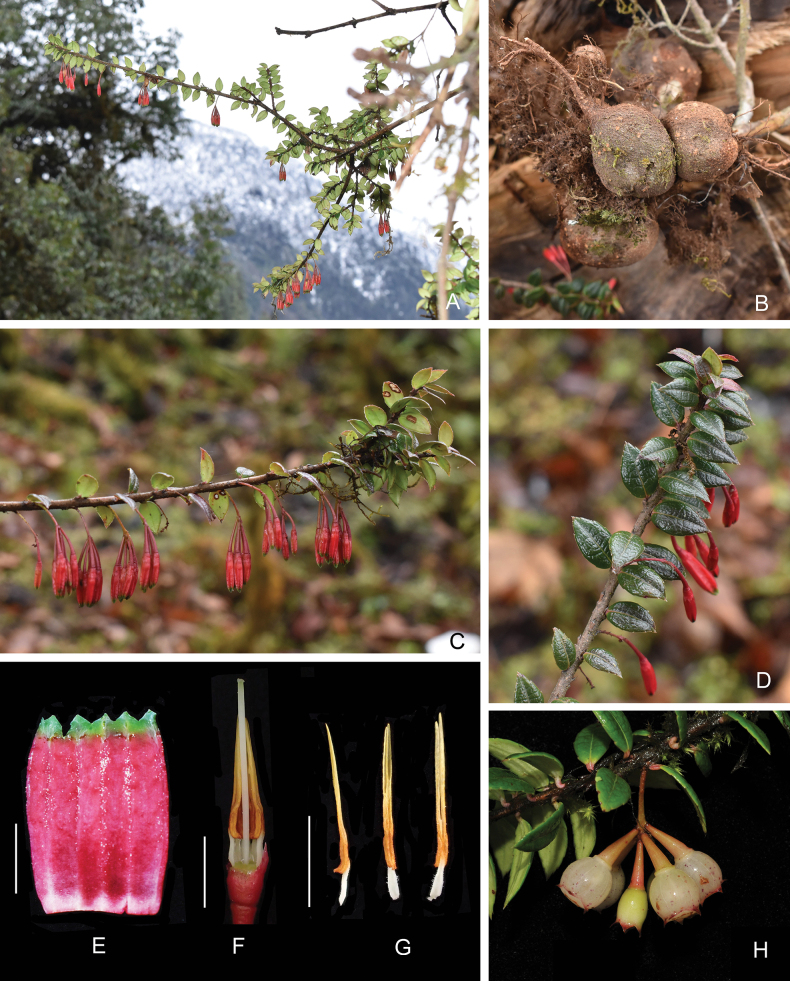
Agapetesforrestiivar.parvifolia**A** habit **B** root tubers **C** flowering branch **D** leafy branch **E** opened corolla, abaxial view **F** flowers with corolla and some stamens removed to show style **G** stamens, lateral (left), adaxial (middle) and abaxial (right) view **H** infructescence. Scale bars: 5 mm (**E–G**). All by Yi-Hua Tong, except **H** by Jie Cai.

#### Diagnosis.

This new variety differs from the nominate variety in having smaller leaves (1.1–1.8 × 0.5–0.7 cm) with an acute apex, nearly transverse secondary veins, puberulent peduncle and shorter corollas (9–12 mm).

#### Etymology.

The variety epithet is derived from its small leaves compared with the nominate variety. The Chinese name is given as 小叶伞花树萝卜(Chinese pinyin: xiǎo yè sǎn huā shù luó bo).

#### Distribution and habitat.

This species is currently known only from the type locality, Medog County, Xizang, China. It grows on the tree trunks under broadleaved forests at elevations of 2190–2700 m.

#### Phenology.

The new variety flowers in November to January the next year and fruits in May to July.

#### Additional specimens examined.

(paratypes): China. Xizang Autonomous Region: Medog County: Damu Xiang, Km 54 on Zhamo Road, epipetric; 29°41′31.77″N, 95°31′00.6″E; 2337 m elev.; 17 November 2016 (fl.); *Yong-Jie Guo, Qiao-Rong Zhang, Shao-Fa Qin, Feng-Qiong Zhang, Sangjie Pingcuo 16CS14481* (KUN, barcode no. 1448873) • ibid., Km 74 on Zhamo Road; 29°41′34.88″N, 95°31′1.96″E; 2380 m elev.; 30 May 2013 (fr.); *Jie Cai, En-De Liu, Yong-Jie Guo 13CS7653* (KUN, barcode no. 1375387) • ibid., Km 80 on Zhamo Road; 4 July 2013 (fr.); *Yi-Hua Tong XZ060* (IBSC) • Dexing Xiang, Lage to Hanmi; 18 October 2012 (fl. bud); *Yarlung Zangbo Expedition Team 934* (BJM, barcode no. 0230310, IBSC) • Jialasa Xiang, Gudeng Gongshe; 2300 m elev.; 11 December 1982 (fl.); *Bo-Sheng Li & Shu-Zhi Cheng 2100* (PE, barcode nos. 01907986 & 01907987) • Pangxin District, west bank of Yarlung Zangbo; 2700 m elev.; 15 December 1982 (fl.); *Bo-Sheng Li & Shu-Zhi Cheng 3389* (PE, barcode nos. 0191009 & 0191010).

#### Taxonomic notes.

This new variety looks very much like a smaller version of the nominate variety. Although the nominate variety is also distributed in Medog County (Huang 1986), its distribution area is a little more south compared to that of A.forrestiivar.parvifolia, and no overlapping area was found (Fig. 2). This species also has potential ornamental values, due to its showy carmine corollas with short green lobes at apex.

## Supplementary Material

XML Treatment for Agapetes interdicta

XML Treatment for Agapetes interdicta var. interdicta

XML Treatment for Agapetes interdicta var. flaviflora

XML Treatment for Agapetes forrestii

XML Treatment for Agapetes forrestii var. forrestii

XML Treatment for Agapetes forrestii var. parvifolia

## ﻿Additional information

### Author ORCIDs

Yi-Hua Tong https://orcid.org/0000-0002-5034-005X

Yong-Jie Guo https://orcid.org/0000-0003-1752-4495

### Data availability

All of the data that support the findings of this study are available in the main text.

## References

[B1] Christensen KI (1987) Taxonomic revision of the *Pinusmugo* complex and P.×rhaetica (P.mugo×sylvestris) (Pinaceae). Nordic Journal of Botany 7: 383–408. 10.1111/j.1756-1051.1987.tb00958.x

[B2] Evans WE (1927) Some interesting and undescribed Vacciniaceae from Burma and Western China. Notes from the Royal Botanic Garden Edinburgh 15: 199–208.

[B3] Fang RZ Stevens PF (2005) *Agapetes*. In: Wu ZY Raven PH Hong DY (Eds) Flora of China (Vol.14). Science Press, Beijing & Missouri Botanical Garden Press, St. Louis, 504–517.

[B4] Huang SH (1986) *Agapetes*. In: Wu CY (Ed.) Flora Xizangica (Vol.3). Science Press, Beijing, 706–721.

[B5] Pei XX Chen ZZ Li Q Li XY Pu CZ Luo K Luo J Pu MJ Wang HJ Khanal L Jiang XL (2024) A new species of the genus *Soriculus* (Soricidae, Eulipotyphla, Mammalia) from Medog in the eastern Himalaya. ZooKeys 1195: 139–155. 10.3897/zookeys.1195.115699 38525353 PMC10958163

[B6] POWO (2024) Plants of the World Online. http://powo.science.kew.org/taxon/41044-1 [accessed 1 August 2024]

[B7] Song ZK Zhu AH Liu ZD Qu Z Li Y Ma HX (2022) Three new species of *Hypoxylon* (Xylariales, Ascomycota) on a multigene phylogeny from Medog in Southwest China. Journal of Fungi 8: art. no. 500. 10.3390/jof8050500 PMC9146989 35628755

[B8] Tong YH (2014) A Systematic Study of the Genus *Agapetes* D. Don ex G. Don (Ericaceae). PhD Dissertation, South China Botanical Garden, Chinese Academy of Sciences.

[B9] Tong YH (2016) *Agapetesxiana* sp. nov. (Ericaceae) from Xizang, China. Phytotaxa 252: 289–292. 10.11646/phytotaxa.252.4.6

[B10] Tong YH Wang BM Xia NH (2019) *Agapetesyingjiangensis* (Ericaceae), a new epiphytic species of A.ser.Longifiles from Yunnan, China. Nordic Journal of Botany: e02171. 10.1111/njb.02171

[B11] Tong YH Zhao WL Wang BM Liu ED Cai J Guo YJ (2021) *Vacciniummotuoense* (Ericaceae), a new species from Xizang, China. PhytoKeys 181: 105–111. 10.3897/phytokeys.181.71522 34611456 PMC8448724

[B12] Watthana S (2015) Ericaceae. In: Newman M Barfod A (Eds) Flora of Thailand (Vol.13(part 1)). The Forest Herbarium, Department of National Parks, Wildlife and Plant Conservation, Bangkok, 101–141.

[B13] Yang B Ya JD Tong YH Wang PY Liu C Zhao WL Liu Z Tan YH (2022) *Agapeteshuangiana* (Ericaceae), a new species from Southeast Xizang, China. Taiwania 67: 254–259. 10.6165/tai.2022.67.254

[B14] Yang B Li MK Wang PY Wang BM Zuo YJ Tan YH (2024) *Agapetesrhuichengiana* (Ericaceae), a new species from Southeast Xizang, China. Taiwania 69: 16–19. 10.6165/tai.2024.69.16

